# Hepatic Impairment as a Risk Factor for Drug Safety: Suitability and Comparison of Four Liver Scores as Screening Tools

**DOI:** 10.3390/jcm12216814

**Published:** 2023-10-27

**Authors:** Kathrin Golla, Andreas Benesic, Hanna Mannell, Tobias Dreischulte, Eva Grill, Dorothea Strobach

**Affiliations:** 1 Doctoral Program Clinical Pharmacy, University Hospital, LMU Munich, Marchioninistr. 15, 81377 Munich, Germany; 2 Hospital Pharmacy, University Hospital, LMU Munich, Marchioninistr. 15, 81377 Munich, Germany; 3 Department of Internal Medicine—Gastroenterology, Krankenhaus GmbH Weilheim-Schongau, Marie-Eberth Str. 6, 86956 Schongau, Germany; 4 Department of Physiology, Institute for Theoretical Medicine, University of Augsburg, 86159 Augsburg, Germany; 5 Institute of General Practice and Family Medicine, University Hospital, LMU Munich, Pettenkoferstr. 8a, 80336 Munich, Germany; 6 Institute for Medical Information Processing, Biometrics and Epidemiology, LMU Munich, Marchioninistr. 15, 81377 Munich, Germany

**Keywords:** hepatic impairment, drug safety, screening tools, clinical pharmacist, patient safety

## Abstract

Hepatic impairment (HI) influences the pharmacokinetics and pharmacodynamics of drugs and represents an important risk factor for drug safety. A reliable screening tool for HI identification at hospital admission by pharmacists would be desirable but is currently lacking. Therefore, we tested four liver scores as potential screening instruments. We retrospectively recorded liver/bile diagnoses, symptoms and abnormalities (summarized as hepatic findings) of 200 surgical patients followed by an assessment of the relevance of these findings for drug therapy (rating). The agreement between the Model of Endstage Liver Disease (MELD), Non-alcoholic fatty liver disease fibrosis score (NFS), Fibrosis 4 index (FIB-4), and aspartate-aminotransferase to platelet ratio index (APRI) and the rating was quantified by Cohen’s Kappa. The performance of the scores in this setting was further evaluated by their sensitivity, specificity, positive predictive value (PPV), and negative predictive value (NPV). Of 200 patients, 18 (9%) had hepatic findings relevant for drug therapy. Fair agreement was found for FIB-4 and MELD and slight agreement for APRI and NFS compared to the rating. The highest values for sensitivity, specificity, PPV, and NPV were 41.2% (MELD), 99.3% (APRI), 66.7% (APRI), and 93.6% (MELD), respectively. Due to low performance, none of the scores can be recommended for clinical use as a single screening tool for HI at hospital admission.

## 1. Introduction

Hepatic impairment (HI) leads to significant alterations in the pharmacokinetics and pharmacodynamics of drugs. Depending on the type and severity of the underlying liver disease, metabolism, including the first pass effect, or elimination of drugs may be affected [1,2,3]. In addition, HI can cause a variety of pathophysiologic conditions such as renal dysfunction [4,5] or gastrointestinal bleeding [6]. Accordingly, HI has been identified as a risk factor for drug safety, leading to adverse drug reactions (ADR) [7,8,9]. To prevent ADR, drug therapy needs to be reviewed in terms of drug choice and dose adjustment. To reduce the risk of ADR, patients with HI and at risk for hepatic drug-related problems (DRP) first need to be identified. A pharmacist-led medication reconciliation (PhMR) at hospital admission, including a check for drug adjustments to a patient´s liver function, could help to achieve this aim. A DRP is defined as “an event or circumstance involving drug therapy that actually or potentially interferes with desired health outcomes” [10]. They concern treatment effectiveness or treatment safety and have a variety of potential causes, like incorrect drug selection, drug form, dose selection, or treatment duration [10]. Unfortunately, a suitable, preferably automatic, and reliable screening method to be used by pharmacists to identify patients at risk for hepatic DRP at hospital admission has neither been established nor validated.

To rapidly screen for patients at risk, pharmacists have to rely on documented liver and bile diagnoses or liver laboratory parameters (LLP). However, there are several issues with this approach. First, at hospital admission, patient documentation is often incomplete [11,12,13]. Second, chronic liver disease is judged to be underestimated [14] and about 50% of cirrhosis cases are first diagnosed upon emergency hospitalization [15]. Third, the assessment of liver function and identification of HI based on LLP is still challenging since a single parameter reflecting the complete physiology of the liver does not exist. LLP can be influenced by multiple extrahepatic factors and not all liver diseases result in LLP alterations [16]. Thus, their use as a screening tool for impaired liver function is limited.

Liver scores, combining different laboratory parameters, symptoms or comorbidities, display another option to assess liver function or liver remodeling. Originally developed by Child et al. [17] and modified by Pugh et al. [18], the Child–Pugh Score (CPS) can be used to assign patients into classes A, B, and C depending on the severity of liver cirrhosis [19]. Beside the laboratory parameters albumin, International Normalized Ratio (INR) or prothrombin time and bilirubin, CPS calculation also requires the assessment of two symptoms, ascites and encephalopathy, which may lead to varying results due to subjective assessment [19], and makes it impossible to calculate the CPS automatically or by pharmacists. Irrespective of this, the Food and Drug Administration (FDA) and the European Medicines Agency (EMA) both recommend the CPS as a basis for the assessment of HI in pharmacokinetic studies from which dosage recommendations can be derived [20,21].

The Model of Endstage Liver Disease score (MELD), calculated from bilirubin, INR and serum creatinine, has been used by the Organ Procurement and Transplantation Network (OPTN)/United Network for Organ Sharing for the allocation of liver donations [22,23]. Since July 2023, the so-called MELD 3.0 has been used for this purpose [24,25]. In contrast to the CPS, the automatic calculation of the MELD is possible since it is based on laboratory parameters only. Additionally, Albarmawi et al. found a good correlation between the CPS and the MELD, thus, the MELD might also be converted to the CPS as a basis for drug adjustment to liver function [26].

The non-alcoholic fatty liver disease (NAFLD) fibrosis score (NFS) is calculated from the laboratory parameters aspartate-aminotransferase (AST), alanine-aminotransferase (ALT), platelet count and albumin, the Body Mass Index (BMI), age, and the impaired fasting glucose/diabetes status. It was originally developed and validated to classify patients with confirmed NAFLD for advanced/not advanced fibrosis [27]. The Fibrosis 4 index (FIB-4) is used for the prediction of fibrosis in patients with human immunodeficiency virus/hepatitis C virus (HCV) coinfection. The FIB-4 can be calculated by the routinely collected parameters AST, ALT, platelet count and age [28]. The AST to platelet ratio index (APRI) has been developed for the prediction of advanced fibrosis and cirrhosis in patients with chronic HCV infection. The actual AST, upper limit of normal of AST and platelet count are required for the calculation [29]. Beside the validated application areas, NFS, FIB-4, and APRI were tested as predictive models for the occurrence of cirrhosis in the general population [30], esophageal varices in hepatocellular carcinoma (HCC) patients [31], as well as mortality among COVID-19 patients [32] or inflammation in drug-induced liver injury [33].

The actual impact of a liver disease on the pharmacokinetics and pharmacodynamics of drugs depends on the kind and severity of hepatic changes. Several mechanisms may be involved. In particular, Cytochrome P450 (CYP) enzymes, which are the major contributors to phase-1 metabolism [34] and represent the predominant enzymes involved in the metabolism of drugs [35], may be influenced by the type and severity of the liver diseases [36,37,38,39,40]. Regarding phase-2 reactions, especially glucuronidation, it can be assumed that these are largely preserved in mild liver diseases [41]. Beside drug-metabolizing enzymes, hepatic transporters like organic anion transporters contribute to the clearance of drugs. Effects on hepatic transporter expression have been described for several liver diseases such as NASH or primary biliary cholangitis. When assessing pharmacokinetic changes in liver disease, this aspect must also be taken into account, since, among other things, altered systemic drug levels may occur [42]. In addition, renal or gastrointestinal transporters may also be affected by hepatic dysfunction [43]. Cirrhosis of the liver can also lead to an impaired blood flow through the liver, e.g., due to the formation of portosystemic shunts, which can cause changes in the bioavailability of drugs [44]. Some liver diseases may be clinically relevant, e.g., liver metastases or HCC, but they might have only a minor impact on the pharmacokinetics of drugs without concomitant cirrhosis [2]. Other liver irregularities, such as small liver cysts or hemangioma, may not have any clinical relevance at all [45]. Especially diseases like NAFLD, which may proceed to non-alcoholic steatohepatitis (NASH), possibly combined with fibrosis, or even cirrhosis [46], are of special interest from a pharmacokinetic point of view, as effects on drug-metabolizing enzymes seem to increase in parallel with the disease progression [36]. In addition, NAFLD is a widespread liver disease, affecting 30% of the worldwide population [47]. Moreover, the prevalence of NAFLD and its advanced stage NASH is expected to rise in the coming years [48].

Taken together, the appropriate identification of patients at risk for hepatic DRP by pharmacists is still challenging. Previously, we studied the MELD as a feasible screening tool for HI to be used by pharmacists at hospital admission. However, certain conditions such as interfering factors and the correct adjustment of MELD parameters to standard values must be taken into account. Moreover, the availability of bilirubin values was a limiting factor [49]. Other established liver scores might represent useful and feasible alternatives.

Thus, the aim of our study was to investigate and compare the suitability of the liver scores MELD, NFS, FIB-4, and APRI as general screening tools for HI by pharmacists in a retrospective surgical patient cohort. We performed interrater reliability analyses and calculated the sensitivity, specificity, positive predictive value (PPV) and negative predictive value (NPV) of the scores in terms of the identification of patients with liver or bile diagnoses, symptoms and abnormalities relevant for drug therapy. To our knowledge, this is the first study testing four different liver scores as potential screening tools for HI to be implemented in clinical routine. 

## 2. Materials and Methods

We performed a retrospective chart review including the first 200 patients aged ≥18 years, who were admitted to the surgical department (including general, visceral, transplant, vascular surgery) of a large university hospital in Bavaria, Germany from March 2021 on and for whom PhMR at hospital admission was performed. Prior to this, a sample size calculation was conducted giving a minimum sample size of 186 patients under the assumption of a 45% dropout rate [50,51]. PhMR is routinely carried out from Monday to Friday and includes a detailed acquisition of prescribed, recently terminated and over-the-counter drugs. For each patient, laboratory parameters from the day of hospital admission, documented liver or bile diagnoses, symptoms or abnormalities and the diabetes status were recorded from the electronic patient information system (SAP-i.s.h.med, Cerner Corporation, North Kansas City, MO, USA). Documented laboratory parameters included ALT, AST, bilirubin, albumin, INR, platelet count, and serum creatinine. The study was performed in accordance with the Declaration of Helsinki. Ethics approval was obtained by the ethics committee of the University Hospital Munich (23-0186).

In an initial step, all confirmed liver and bile diagnoses, and all associated symptoms and structural or functional abnormalities of the liver, the gall bladder and bile ducts were recorded. These were summarized as “hepatic findings”. For this purpose, medical reports, discharge letters and findings of imaging techniques (computer tomography, sonography, magnet resonance imaging) were retrieved from the electronic patient documentation. Current and past hepatic findings were considered as well as those with an unknown status. For patients after liver resection or with evidence (current or past) of liver metastases, HCC, cholangiocellular carcinoma, hepatitides, cirrhosis, fibrosis, fatty liver or NASH, histological findings were searched and reviewed for fibrotic or cirrhotic changes. In patients with advanced fibrosis or cirrhosis, particular attention was paid to findings suggesting portal hypertension, such as ascites or collateral circulation. In a second step, the hepatic findings collected were assessed in terms of general relevance and relevance for drug therapy. The assessment was performed by an interprofessional team consisting of a hepatologist and two pharmacists and was based on medical and pharmacological considerations. The general clinical relevance of hepatic findings was assessed by an accomplished specialist in hepatology. Evaluation regarding the impact of hepatic findings on drug therapy was followed by a thorough consideration of the possible impact on pharmacokinetic and pharmacologic aspects. The aim of the assessment was to assign one of three possible categories to each previously identified hepatic finding in terms of its general relevance and relevance for drug therapy:

(1) Relevant (hepatic finding has a general relevance or relevance for drug therapy without further assessment of the patient case);
(2) Irrelevant (hepatic finding has no general relevance or relevance for drug therapy without further assessment of the patient case);
(3) Patient individual judgement (to decide whether the hepatic finding has a general relevance or a relevance for drug therapy, a more in-depth consideration of the patient’s case must be made, taking into account the underlying disease, manifestation, current status, current symptoms, and severity of the hepatic finding).

The results of the interprofessional discussion were tabulated and served as a basis for the subsequent rating for the relevance of hepatic findings. Rating 1: assessment of all patients regarding general relevance of hepatic findings. Rating 2: assessment of all patients regarding relevance of hepatic findings for drug therapy. For this purpose, patients were assigned to the following categories: “Patients with relevant hepatic findings”, “Patients with hepatic findings relevant for drug therapy” and “Patients with hepatic findings not relevant”. The assignment into these categories was based on the current hepatic findings. The presence of hepatic findings with an unknown status and/or past hepatic findings only led to an assignment into the category “Patients with hepatic findings not relevant”. The only exceptions were conditions after liver transplantation and liver resection, which were recorded as past but considered and evaluated as current findings.

**Figure 1:**
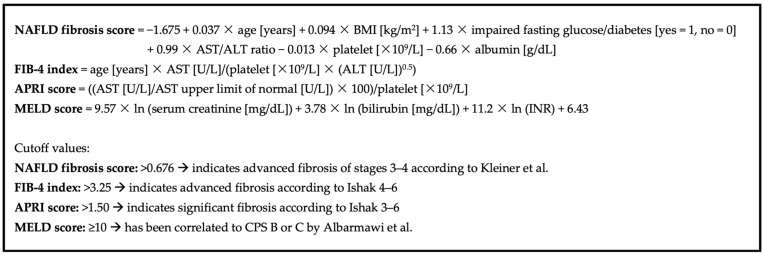
Score formulas and cutoff-values [22,26,27,28,29,52,53].

The MELD, NFS, APRI and FIB-4 were calculated for all patients with the parameters available at hospital admission. HI possibly relevant or relevant for drug therapy is expected for patients with CPS-B or C or an advanced fibrosis, thus, corresponding cutoff-values of the scores were used according to recommendations in the literature. The threshold for the MELD was set at 10 as MELD ≥ 10 has been correlated to CPS of B or C, indicating moderate to severe hepatic insufficiency [26]. MELD parameters were adjusted according to previous OPTN policies (effective date: 18 June 2020) prior to calculation [22]. The cutoff value for the NFS was accepted as >0.676 as this indicates an advanced fibrosis in NAFLD-patients according to Kleiner et al. [27,52]. In the original formula, the presence of abnormal fasting blood glucose levels or diabetes is considered [27]. Since it is not possible to determine if blood sampling was performed in fasting patients, only diabetes was included in the NFS calculation. For the FIB-4, the reported cutoff value of >3.25 for advanced fibrosis corresponding to Ishak 4–6 was used [28,53]. Wai et al. have determined a fibrosis cutoff value of >1.50 for the APRI, indicating a significant fibrosis according to Ishak 3–6, which was also accepted for this study [29,53]. Formulas and respective cutoff values are shown in Figure 1. Since the considered thresholds of the FIB-4, APRI, and NFS indicate significant fibrosis, the presence of low-grade or mild fibrosis was not considered an indicator for HI.

Continuous variables are presented with median and range, and categorical variables as frequency distribution. To assess the interrater reliability of the scores, the Kappa coefficient with related 95% confidence interval (95% CI) was calculated. Cohen´s Kappa was calculated for pairwise interrater reliability between the MELD, NFS, FIB-4 and APRI and each of the scores and Rating 1 and Rating 2. Fleiss´ Kappa was applied for the determination of interrater reliability between all scores. Statistical significance was accepted as *p* < 0.05. In order to correct for multiple testing, *p*-values were adjusted according to Holm–Bonferroni. The interpretation of Kappa followed the agreement classification of Landis and Koch [54]. Sensitivity, specificity, PPV, and NPV were calculated for each score regarding the identification of patients with hepatic findings relevant for drug therapy. Data were documented with Microsoft Excel^®^ 2016 (Seattle, WA, USA) and statistical analyses were performed with IBM SPSS Statistics^®^ version 29.0 (Armonk, NY, USA).

## 3. Results

**Figure 2:**
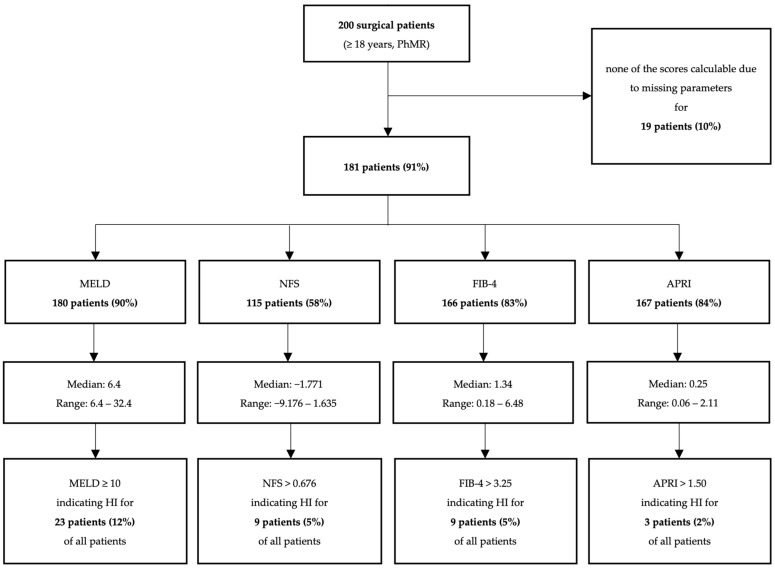
Calculation of liver scores for 200 surgical patients at hospital admission and identification of patients with potential hepatic impairment (HI).

Of the 200 patients, 102 (51%) were female and the median age was 62 years (20–86 years). For 115 (58%) patients, all four scores could be calculated. In detail, out of 200 patients, the MELD, APRI, FIB-4, and NFS were calculable for 180 (90%), 167 (84%), 166 (83%) and 115 (58%) patients, respectively. The most frequently missing parameters were bilirubin for the MELD (19, 10%), albumin for the NFS (80, 40%) and AST for the APRI and the FIB-4 (33, 17%). In comparison to the other scores, the MELD most frequently indicated a possible HI. Related to all 200 patients, the MELD indicated an HI for 12%, the APRI for 2% and the NFS and the FIB-4 for 5% of patients (Figure 2). Referring only to those patients, for whom a calculation of the respective score was possible, this corresponds to MELD, APRI, NFS, and FIB-4 indicating a possible HI in 13%, 2%, 8%, and 5% of patients, respectively (Table 1).

### 3.1. Categorisation of Hepatic Findings

A total of 548 hepatic findings were documented with at least one current, past or unknown status for 145 (73%) of the 200 screened patients. Of the 548 hepatic findings, 317 (58%) were categorized as current, 188 (34%) as past and 43 (8%) as unknown status. Overall, 76 different hepatic findings were identified and assessed by the interprofessional team (Table 2).

Of the 145 patients with a documented hepatic finding, 62 (43%) were classified as “Patients with a relevant hepatic finding”. For 18 (29%) of these patients, corresponding to 9% of all screened 200 patients, at least one hepatic finding was considered to probably have an impact on drug therapy. Hepatic findings were assessed as not relevant for 83 (57%) of the 145 patients (Table 1). At least one hepatic finding of unknown status was noted in 29 of 145 (20%) patients, including 26 patients with at least one additional current hepatic finding. Accordingly, 62 patients in Rating 1 had relevant hepatic findings and 138 patients irrelevant or no hepatic findings. In Rating 2, 18 patients had hepatic findings relevant for drug therapy, and 182 patients had hepatic findings not relevant for drug therapy or no findings.

### 3.2. Overall Interrater Reliability of Hepatic Scores

Testing for interrater reliability by Fleiss´ Kappa calculation between MELD, NFS, APRI and FIB-4 indicating a potential HI was possible for 115 patients. A Kappa value of 0.172 (95% CI 0.098–0.246, *p* < 0.001, *p*-value adjusted according to Holm–Bonferroni pB < 0.001) was calculated and interpreted as a slight agreement.

### 3.3. Pairwise Interrater Reliability of Hepatic Scores and Ratings

Next, we performed pairwise tests for interrater reliability between the four scores, and between the scores and the ratings. The results are presented in Table 3. Calculation between the MELD and the NFS was possible for 115 patients, the MELD and the FIB-4 for 166, the MELD and the APRI for 167, the NFS and the FIB-4 for 115, the NFS and the APRI for 115, and the FIB-4 and the APRI for 166 patients, respectively. The interrater reliability between the FIB-4 and the NFS reached moderate agreement. The pairwise agreements between the remaining scores were classified as slight for the MELD and the NFS, the MELD and the FIB-4, the MELD and the APRI, and the APRI and the FIB-4, and as poor for the NFS and the APRI.

Pairwise interrater reliability between the scores MELD, FIB-4, APRI, NFS and Ratings 1 and 2 was calculable for 180, 166, 167 and 115 patients, respectively. Fair agreement was found between Rating 2 and the MELD and FIB-4. Slight agreement was determined for the NFS and APRI and Rating 2 (Table 3). The Kappa values for interrater reliability indicate a slight agreement between each of the scores MELD, FIB-4 and APRI and Rating 1. For the NFS and Rating 1, poor agreement was found (Table 3).

### 3.4. Sensitivity, Specificity, Positive Predictive Value, and Negative Predictive Value

Due to differences in the availability of the four scores tested, the calculations were performed in 180 cases for the MELD, 167 for the APRI, 166 for the FIB-4, and 115 for the NFS. The MELD had the highest values for sensitivity (41.2%) and the NPV (93.6%), and APRI had the highest values for specificity (99.3%) and PPV (66.7). The results for the scores used as screening tools for patients with hepatic findings relevant for drug therapy are presented in Table 4.

## 4. Discussion

To our knowledge, this is the first study testing the suitability of four different established liver scores as possible screening tools for hepatic impairment at hospital admission by pharmacists. In our retrospective surgical patient cohort, the number of patients identified to have possible HI differed widely from 13% by the MELD to 8% by the NFS, 5% by the FIB-4, and 2% by the APRI. Interrater reliability between the scores was only poor or slight, with the exception of a moderate agreement between the FIB-4 and the NFS. Likewise, only poor or slight agreement was found between the tested scores and the categorization of patients as having relevant hepatic findings or not. However, for the assessment of patients regarding relevance of hepatic findings for drug therapy, fair agreement was found for the FIB-4 and MELD, and slight agreement for the NFS and APRI. In addition, for all scores, values of 90.2% and higher were reached for specificity and the NPV, whereas the highest values for sensitivity and the PPV were only 41.2% (MELD) and 66.7% (APRI) regarding the identification of patients with hepatic findings relevant for drug therapy. Moreover, the study underlines the diversity of liver and bile diseases and symptoms, and questions how these can be represented by the considered liver scores. In sum, none of the scores tested in this study achieved excellent results and can thus be recommended to be used as an isolated screening tool to identify patients with HI at risk for hepatic DRP by pharmacists.

As described, only a slight overall agreement was found between the investigated scores regarding screening for HI. A possible explanation may be found in the different parameters of the scores, reflecting various etiologies of liver diseases. In fact, all scores have been developed for special hepatic patient groups [27,28,29,55,56], which might lead to the under- or overestimation of the clinical status of a patient when applying the scores for other etiologies. However, the use of these scores in different settings has been tested by others [30,31,32,33,57].

In addition, the results regarding the identification of patients with liver or bile diagnoses, symptoms or abnormalities relevant for drug therapy were unsatisfying. Compared to all scores, the greatest agreement with the assessment of patients regarding these hepatic findings was found for the MELD and FIB-4 (fair agreement). However, in the clinical settings, low agreement levels are considered unsatisfactory and not safe [58]. The sensitivity and specificity of the scores also highlight that none of the scores can be applied without restrictions. Although specificity values > 90% were calculated for each of the scores, the sensitivity calculations with a maximum value of 41.2% for the MELD show that only an insufficient number of patients with an HI relevant for drug therapy can be identified. Furthermore, none of the scores reflect the complex pathological changes affecting the pharmacokinetics of drugs.

The calculation of the scores was limited by the availability of parameters. In this study, the MELD, the APRI, and the FIB-4 could be calculated for the majority of patients, whereas the NFS was only available for 58% of patients. While the MELD, APRI and FIB-4 can be calculated automatically, NFS calculation requires knowledge of the diabetic status, which can only be obtained by screening the patient’s file. Alternatively, fasting blood glucose levels are needed, but the timing of standard blood sampling regarding meals is usually unknown and glucose levels in laboratory data of little benefit. Therefore, the NFS calculation is more time-consuming compared to the other scores. In addition, interfering factors of parameters of the MELD [49], the APRI, the FIB-4 and the NFS [59] might impact automatic interpretation.

Due to the broad variety of liver and bile diseases, as also seen in this study, a differentiated view on the potential effects on drug therapy is required, and the classification of liver and bile diseases relevant for drug therapy remains challenging. Therefore, the subdivision of hepatic findings made here can be questioned, especially since it strongly depends on the completeness of medical records and subjective clinical judgement. However, our ratings were based on an in-depth interprofessional discussion considering the possible effects of hepatic findings on pharmacokinetics, pharmacodynamics, and the choice of drugs. Nevertheless, there is still uncertainty regarding the effects of several hepatic findings. As an example, it remains challenging to assess the effects that HCC and fatty liver may have on CYP enzymes [36,40]. In addition, pathological changes following severe liver disease, like portal hypertension, ascites or hepatic encephalopathy, have to be considered for drug choice and dosage to prevent DRP and adverse effects in hepatic patients [60].

Moreover, alternative screening tools, such as screening for elevated LLP and ALT or the use of analytical CPS (considering laboratory parameters of CPS only) as tested by our group and others, did not perform well in the identification of patients at risk, and may under- or overestimate the number of patients with HI [49,61,62]. Considering this and the results of our study presented here, we conclude that a safe identification of patients suffering from HI at hospital admission can currently only be ensured by a trained pharmacist who is able to identify all patient-specific aspects related to a possible HI. Nevertheless, an initial automated screening using liver scores or other easily accessible parameters would still be desirable, and could facilitate the identification of patients at hepatic risk. It remains to be seen whether a combination of different scores or a solution involving artificial intelligence will lead to a more reliable assessment. Regardless of its insufficient performance in this study, the MELD should be emphasized, as it achieved the highest sensitivity, and along with the FIB-4 a fair agreement regarding the identification of patients with hepatic findings relevant for drug therapy. In addition, its parameters were available for most patients. Moreover, the MELD can be linked to the CPS [26], which is frequently used for drug adjustment to liver function following recommendations by the EMA and FDA [20,21]. An automatic MELD calculation for all inpatients by clinical decision support systems has indeed been suggested [57]. However, our study suggests that the use of the MELD as an indicator for HI cannot be recommended. Interestingly, a different version of the MELD has been recently incorporated into the OPTN guidelines for prioritization in liver allocation, including bilirubin, INR, serum creatinine, serum sodium, albumin and sex [24,25]. It remains to be seen whether this score is advantageous in the identification of patients with HI and in need of drug adjustment compared with the MELD considered in this study.

The lack of verification of the accuracy of the interprofessional assessment of the hepatic findings themselves, or rather the lack of verification of the accuracy of the rating of patients based on this assessment, is the key limitation of this study. Especially the rating of patients requiring an individual patient judgement depends on the experience level of the pharmacist or physician, and can be biased. However, as there is neither an established and validated guideline nor a tool to identify patients at hepatic risk to this date, our approach of assessing hepatic findings interprofessionally before rating the patients seemed to be the best currently available option, as it reflects the pharmaceutical and hepatological point of view. The study is further limited by its retrospective design, the availability of laboratory parameters and the incomplete documentation of liver or bile diagnoses, symptoms or abnormalities. Hepatic findings like “fatty liver” are often mentioned but poorly characterized, thus, their possible impact on drug therapy remains unclear. In addition, missing knowledge of patients´ liver disease is a known clinical problem [14,63]. A routinely performed screening for hepatic impairment by a trained pharmacist may help to improve this matter. Laboratory parameters of the scores can be influenced by multiple extrahepatic reasons, which could lead to false alerts suggesting hepatic impairment [49,59]. Moreover, the age might have an impact on the reliability of some scores. Thus, the use of the NFS and the FIB-4 in NAFLD-patients ≤ 35 years, for example, has been questioned [64]. In this study, the cutoff values of the original publications were applied, but the use of alternative thresholds can be discussed as well. For instance, optimized thresholds for the FIB-4 indicating or excluding significant fibrosis in NAFLD patients [65] and alternative cutoff values for the APRI in patients with HCV infection [66] have been published.

## 5. Conclusions

To summarize, the single use of the MELD, NFS, APRI or FIB-4 cannot be recommended as suitable screening tools for the safe identification of patients with hepatic impairment by pharmacists at hospital admission. Currently, reliable identifications of patients can only be facilitated by a trained pharmacist and an intensive review of the patient’s case, as performed in this study. It remains to be seen whether a combination of different scores will lead to a more reliable identification of patients at risk for hepatic drug-related problems. Further prospective studies testing alternative screening instruments for this purpose would therefore be desirable.

## Figures and Tables

**Table 1:** Calculation of liver scores for predefined groups.

	Overall	Patients without Hepatic Findings	Patients with Hepatic Findings (Current, Past or Status Unknown)	Patients with Hepatic Findings Not Relevant	Patients with Relevant Hepatic Findings	Patients with Hepatic Findings Relevant for Drug Therapy
No. patients	200	55	145	83	62	18
Female (*n*, %)	102 (51%)	27 (49%)	75 (52%)	51 (61%)	24 (39%)	7 (39%)
Age (years)	62 (20–86)	52 (20–86)	64 (22–84)	64 (23–84)	64 (22–83)	62 (38–79)
MELD						
Available (*n*, %)	180 (90%)	44 (80%)	136 (94%)	77 (93%)	59 (95%)	17 (94%)
Median	6.4	6.4	6.4	6.4	6.4	8.5
Range	6.4–32.4	6.4–18.1	6.4–32.4	6.4–32.4	6.4–19.7	6.4–19.7
Indicating HI (*n*, %) ^a^	23 (13%)	5 (11%)	18 (13%)	10 (13%)	8 (14%)	7 (41%)
NFS						
Available (*n*, %)	115 (58%)	32 (58%)	83 (57%)	46 (55%)	37 (60%)	12 (67%)
Median	−1.771	−1.900	−1.762	−1.689	−1.762	−0.547
Range	−9.176–1.635	−6.889–1.635	−9.176–1.592	−9.176–1.566	−8.631–1.592	−4.430–1.592
Indicating HI (*n*, %) ^a^	9 (8%)	2 (6%)	7 (8%)	5 (11%)	2 (5%)	2 (17%)
APRI						
Available (*n*, %)	167 (84%)	39 (71%)	128 (88%)	69 (83%)	59 (95%)	16 (89%)
Median	0.25	0.23	0.26	0.27	0.24	0.74
Range	0.06–2.11	0.11–1.27	0.06–2.11	0.13–1.47	0.06–2.11	0.10–2.11
Indicating HI (*n*, %) ^a^	3 (2%)	0	3 (2%)	0	3 (5%)	2 (13%)
FIB-4						
Available (*n*, %)	166 (83%)	39 (71%)	127 (88%)	68 (82%)	59 (95%)	16 (89%)
Median	1.34	1.01	1.40	1.47	1.38	1.56
Range	0.18–6.48	0.18–2.86	0.32–6.48	0.44–5.46	0.32–6.48	0.48–6.48
Indicating HI (*n*, %) ^a^	9 (5%)	0	9 (7%)	4 (6%)	5 (8%)	5 (31%)

^a^ In relation to the number of patients for whom the respective score was available.

**Table 2:** All documented hepatic findings (liver/bile diagnoses/symptoms/abnormalities) of all included patients (*n* = 200). Hepatic findings were subdivided into current, past and status unknown. The last two columns indicate the classification as relevant and relevant for drug therapy, assuming that the hepatic finding is currently present.

Liver or Bile Diagnoses/ Symptoms/ Abnormalities	Overall	Current	Past	Status Unknown	Relevant Hepatic Finding	Hepatic Finding Relevant for Drug Therapy
**Cholestasis-associated hepatic findings ^a^**	68	37	24	7	PIJ	PIJ
Dilatation of bile ducts	26	13	7	6	PIJ	PIJ
Biliary stent	15	10	5	0	PIJ	PIJ
Prominent bile ducts	9	8	0	1	PIJ	PIJ
Stone passage	6	0	6	0	Yes	PIJ
Choledochal stenosis	4	3	1	0	Yes	PIJ
Percutaneous transhepatic cholangiodrainage	3	3	0	0	PIJ	PIJ
Icterus	3	0	3	0	Yes	Yes
Cholestatic itching	1	0	1	0	Yes	Yes
Bile duct obstruction	1	0	1	0	Yes	PIJ
**Small, asymptomatic benign** **neoplasms**	66	65	1	0	No	No
**Cholecystitis**	40	22	16	2	PIJ	No
**Post cholecystectomy**	39	n.a.	39	n.a.	No	No
**Fatty liver**	38	33	0	5	PIJ	PIJ
**Cholecystolithiasis**	34	30	4	0	PIJ	No
**Liver metastases**	26	19	7	0	Yes	PIJ
**Liver fibrosis/Fibrotic changes**	21	15	5	1	PIJ	PIJ
**Cholestasis**	18	7	6	5	Yes	PIJ
**Post liver resection ^b^**	16	n.a.	16	n.a.	PIJ	PIJ
<1 month ago	0	n.a.	0	n.a.	PIJ	PIJ
>1 month ago	16	n.a.	16	n.a.	PIJ	PIJ
**Focal fatty change**	11	11	0	0	No	No
**Gall bladder sludge**	11	8	3	0	No	No
**Ascites**	10	3	5	2	Yes	PIJ
**Bilioma/Bile leakage**	9	4	5	0	Yes	No
**Cholangiocarcinoma**	8	5	3	0	Yes	PIJ
**Hepatomegaly**	6	5	1	0	PIJ	PIJ
**Portal vein stenosis**	6	3	3	0	Yes	PIJ
**Portal hypertension**	6	3	1	2	Yes	Yes
**Gallbladder hydrops**	6	2	2	2	Yes	PIJ
**Cholangitis**	6	1	5	0	Yes	PIJ
**Hepatopathy**	5	3	0	2	Yes	PIJ
**Choledocholithiasis**	5	1	3	1	Yes	PIJ
**Post biliodigestive anastomosis** **formation**	5	n.a.	5	n.a.	No	No
**Prominent lymph nodes porta hepatis**	4	4	0	0	PIJ	No
**NASH**	4	4	0	0	Yes	Yes
**Abnormal lymph nodes liver hilus**	4	3	1	0	PIJ	No
**Hepatocellular carcinoma**	4	3	1	0	Yes	PIJ
**Portal vein thrombosis** **(+/− cavernous transformation)**	4	3	0	1	Yes	PIJ
**Periportal edema**	4	1	1	2	PIJ	PIJ
**Esophageal varices**	4	1	1	2	Yes	Yes
**Hepatitis B**	4	0	3	1	Yes	PIJ
**Biliary pancreatitis**	4	0	4	0	Yes	PIJ
**Fluid retention post liver resection**	3	2	1	0	PIJ	No
**Congested liver**	3	1	0	2	Yes	PIJ
**Liver cirrhosis**	2	2	0	0	Yes	Yes
**Symptomatic liver cyst**	2	2	0	0	Yes	PIJ
**Cholesteatosis**	2	2	0	0	No	No
**Prominent liver lobe**	2	2	0	0	PIJ	No
**Portal-hypertensive gastropathy**	2	2	0	0	Yes	Yes
**Irregularities of liver surface/liver** **capsule**	2	1	0	1	PIJ	PIJ
**Hepatitis A**	2	0	2	0	Yes	PIJ
**Hepatitis without further definition**	2	0	2	0	Yes	PIJ
**Liver abscess**	2	0	2	0	Yes	PIJ
**Hepatitis C**	2	0	2	0	Yes	PIJ
**Hepatosplenomegaly**	2	0	2	0	Yes	PIJ
**Post liver transplantation**	2	n.a.	2	n.a.	PIJ	PIJ
**Symptomatic liver adenoma**	1	1	0	0	Yes	PIJ
**Gallbladder polyp**	1	1	0	0	PIJ	No
**Lipofucinosis of the liver**	1	1	0	0	No	No
**Superinfected necrosis**	1	1	0	0	Yes	PIJ
**Superinfected hematoma**	1	1	0	0	Yes	PIJ
**Liver parenchymal damages**	1	1	0	0	Yes	PIJ
**VRE-detection in bile**	1	1	0	0	Yes	No
**Intrahepatic hemorrhage**	1	0	1	0	Yes	PIJ
**Tumorous gall bladder**	1	0	1	0	Yes	PIJ
**Tumor infiltration of the liver**	1	0	1	0	Yes	PIJ
**Symptomatic polycystic** **liver disease**	1	0	1	0	Yes	PIJ
**Symptomatic intrahepatic bile duct cyst**	1	0	1	0	Yes	PIJ
**Gallbladder carcinoma**	1	0	1	0	Yes	PIJ
**Biliary fistula**	1	0	1	0	Yes	No
**Bile duct injury**	1	0	1	0	Yes	No
**Hepatic encephalopathy**	1	0	1	0	Yes	Yes
**Post portal bifurcation resection**	1	n.a.	1	n.a.	No	No
**Liver vein thrombosis**	1	0	0	1	Yes	PIJ
**Ischemic Type Biliary Lesions**	1	0	0	1	Yes	PIJ
**Liver insufficiency**	1	0	0	1	Yes	Yes
**Collateral circulation**	1	0	0	1	Yes	Yes
**Intrahepatic fluid retention**	1	0	0	1	PIJ	PIJ
**∑ ^c^**	548	317	188	43		

n.a.: not applicable; PIJ: patient individual judgement depending on the underlying disease, manifestation, current status, current symptoms, and severity. ^a^ other causes possible; ^b^ 10 patients post hemihepatectomy, 6 of the 16 patients had both a hemihepatectomy and a partial liver resection; ^c^ the categories “Cholestasis-associated hepatic findings” and “Post liver resection” represent superordinate categories and are therefore not added to the sum.

**Table 3:** Pairwise interrater reliability between MELD, NFS, APRI and FIB-4, and between the scores and Ratings 1 and 2. *p*-values were adjusted according to Holm–Bonferroni (pB). Agreement interpretation followed Landis and Koch.

	MELD	NFS	FIB-4	APRI
NFS	κ = 0.183 (*p* = 0.043, pB = 0.345) (95% CI −0.066–0.432)	-	-	-
FIB-4	κ = 0.142 (*p* = 0.045, pB = 0.345) (95% CI −0.060–0.344)	κ = 0.555 (*p* < 0.001, pB < 0.001) (95% CI 0.261–0.849)	-	-
APRI	κ = 0.053 (*p* = 0.277, pB = 1.000) (95% CI −0.092–0.198)	κ = −0.016 (*p* = 0.770, pB = 1.000) (95% CI −0.043–0.011)	κ = 0.143 (*p* = 0.031, pB = 0.281) (95% CI −0.141–0.427)	-
Rating 1: Identification of patients with relevant hepatic findings	κ = 0.014 (*p* = 0.826, pB = 1.000) (95% CI −0.111–0.139)	κ = −0.045 (*p* = 0.506, pB = 1.000) (95% CI −0.165–0.075)	κ = 0.058 (*p* = 0.197, pB = 1.000) (95% CI −0.040–0.156)	κ = 0.065 (*p* = 0.018, pB = 0.180) (95% CI −0.006–0.136)
Rating 2: Identification of patients with hepatic findings relevant for drug therapy	κ = 0.271 (*p* < 0.001, pB = 0.003) (95% CI 0.067–0.475)	κ = 0.111 (*p* = 0.228, pB = 1.000) (95% CI −0.128–0.350)	κ = 0.355 (*p* < 0.001, pB < 0.001) (95% CI 0.104–0.606)	κ = 0.186 (*p* = 0.001, pB = 0.008) (95% CI −0.049–0.421)

Agreement interpretation according to Landis and Koch [54]: 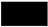 moderate agreement; 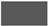 fair agreement; 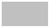 slight agreement; 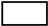 poor agreement.

**Table 4:** Sensitivity, specificity, positive predictive value, and negative predictive value of the tested scores regarding identification of patients with hepatic findings relevant for drug therapy. For the MELD 180 patients, for the NFS 115 patients, for the FIB-4 166 patients, and for the APRI 167 patients were considered. Prevalence indicates the occurrence of patients with hepatic findings relevant for drug therapy in the groups studied.

	MELD	NFS	FIB-4	APRI
Prevalence	9.4%	10.4%	9.6%	9.6%
Sensitivity	41.2%	16.7%	31.3%	12.5%
Specificity	90.2%	93.2%	97.3%	99.3%
Positive predictive value	30.4%	22.2%	55.6%	66.7%
Negative predictive value	93.6%	90.6%	93.0%	91.5%

## Data Availability

The datasets used during the current study are available from the corresponding author on reasonable request.
